# Supplementary material for: ProteinShader: illustrative rendering of macromolecules
Source: BMC Struct Biol. 2009 Mar 30;9:19. doi: 10.1186/1472-6807-9-19 (PMC2672931; doi:10.1186/1472-6807-9-19)
Supplement: Additional file 1 — ProteinShader program without source code. This compressed file contains the complete ProteinShader program including associated libraries, but no source code. A README.txt file gives an overview of the ProteinShader distribution, and the index.html file in the help subdirectory has directions on getting started with the program as well as a set of tutorials. [file 1472-6807-9-19-S1.zip › ProteinShader-beta-0_9_4-binary/help/api/constant-values.html]

Constant Field Values (ProteinShader API)


|  |  |  |
| --- | --- | --- |
| **Overview** \| Package \| Class \| Use \| **Tree** \| **Deprecated** \| **Index** \| **Help** | |  |
| PREV   NEXT | **FRAMES**    **NO FRAMES**     **All Classes** |  |


---


# Constant Field Values


---

**Contents**

- org.proteinshader.\*

org.proteinshader.\*

| org.proteinshader.graphics.Cylinder | | |
| --- | --- | --- |
| `public static final int` | `MIN_CAP_TILING` | `3` |
| `public static final int` | `MIN_SLICES` | `3` |
| `public static final int` | `MIN_STACKS` | `1` |

| org.proteinshader.graphics.FrenetFrames | | |
| --- | --- | --- |
| `public static final double` | `AXIS_RADIUS` | `0.05` |
| `public static final int` | `FRAMES_PER_SEGMENT` | `11` |
| `public static final double` | `MAJOR_AXIS_SCALE` | `1.5` |
| `public static final double` | `MAJOR_SPHERE_RADIUS` | `0.3` |
| `public static final int` | `MAJOR_SPHERE_TILING` | `30` |
| `public static final double` | `MINOR_AXIS_SCALE` | `0.5` |
| `public static final double` | `MINOR_SPHERE_RADIUS` | `0.0375` |
| `public static final int` | `MINOR_SPHERE_TILING` | `10` |
| `public static final int` | `SLICES` | `10` |
| `public static final float` | `SPECULAR_EXP` | `20.0f` |
| `public static final int` | `STACKS` | `1` |
| `public static final double` | `TIP_FRACTION` | `0.2` |
| `public static final double` | `TIP_RADIUS` | `0.08` |

| org.proteinshader.graphics.Ribbon | | |
| --- | --- | --- |
| `public static final double` | `ALPHA_HEIGHT` | `4.0` |
| `public static final float` | `ALPHA_S_COORD_END` | `1.0f` |
| `public static final float` | `ALPHA_S_COORD_START` | `0.0f` |
| `public static final int` | `ALPHA_STACKS` | `10` |
| `public static final float` | `ALPHA_THIN_SIDE_S_MAX` | `0.125f` |
| `public static final float` | `ALPHA_THIN_SIDE_S_MIN` | `0.0f` |
| `public static final double` | `ALPHA_WIDTH` | `0.5` |
| `public static final double` | `BETA_HEIGHT` | `4.0` |
| `public static final float` | `BETA_S_COORD_END` | `1.0f` |
| `public static final float` | `BETA_S_COORD_START` | `0.0f` |
| `public static final int` | `BETA_STACKS` | `10` |
| `public static final float` | `BETA_THIN_SIDE_S_MAX` | `0.125f` |
| `public static final float` | `BETA_THIN_SIDE_S_MIN` | `0.0f` |
| `public static final double` | `BETA_WIDTH` | `0.5` |
| `public static final double` | `LOOP_HEIGHT` | `1.0` |
| `public static final float` | `LOOP_S_COORD_END` | `0.75f` |
| `public static final float` | `LOOP_S_COORD_START` | `0.25f` |
| `public static final int` | `LOOP_STACKS` | `10` |
| `public static final float` | `LOOP_THIN_SIDE_S_MAX` | `0.125f` |
| `public static final float` | `LOOP_THIN_SIDE_S_MIN` | `0.0f` |
| `public static final double` | `LOOP_WIDTH` | `0.25` |

| org.proteinshader.graphics.Shape | | |
| --- | --- | --- |
| `public static final double` | `HALF_PI` | `1.57079632679489` |
| `public static final double` | `PI` | `3.14159265358979` |
| `public static final double` | `TWO_PI` | `6.28318530717958` |

| org.proteinshader.graphics.Silhouette | | |
| --- | --- | --- |
| `public static final boolean` | `DEFAULT` | `true` |

| org.proteinshader.graphics.Sphere | | |
| --- | --- | --- |
| `public static final int` | `MIN_TILING` | `3` |

| org.proteinshader.graphics.Tube | | |
| --- | --- | --- |
| `public static final double` | `ALPHA_RADIUS` | `1.5` |
| `public static final int` | `ALPHA_SLICES` | `20` |
| `public static final int` | `ALPHA_STACKS` | `10` |
| `public static final double` | `BETA_RADIUS` | `1.5` |
| `public static final int` | `BETA_SLICES` | `20` |
| `public static final int` | `BETA_STACKS` | `10` |
| `public static final double` | `LOOP_RADIUS` | `0.75` |
| `public static final int` | `LOOP_SLICES` | `10` |
| `public static final int` | `LOOP_STACKS` | `10` |
| `public static final float` | `S_MAX` | `2.0f` |
| `public static final float` | `S_MIN` | `0.0f` |

| org.proteinshader.graphics.adapter.StructureToGraphics | | |
| --- | --- | --- |
| `public static final boolean` | `AUTO_TILING` | `true` |

| org.proteinshader.graphics.displaylists.CylinderReferences | | |
| --- | --- | --- |
| `public static final double` | `COEFFICIENT` | `29.059` |
| `public static final int` | `DEFAULT_CAP_TILING` | `5` |
| `public static final double` | `DEFAULT_HEIGHT` | `1.0` |
| `public static final double` | `DEFAULT_RADIUS` | `0.15` |
| `public static final int` | `DEFAULT_SLICES` | `10` |
| `public static final int` | `DEFAULT_STACKS` | `1` |
| `public static final double` | `EXPONENT` | `-0.5267` |
| `public static final int` | `MAX_SLICES` | `14` |

| org.proteinshader.graphics.displaylists.SphereReferences | | |
| --- | --- | --- |
| `public static final double` | `COEFFICIENT` | `226.1` |
| `public static final double` | `DEFAULT_RADIUS` | `1.0` |
| `public static final int` | `DEFAULT_TILING` | `12` |
| `public static final double` | `EXPONENT` | `-0.7367` |
| `public static final int` | `MAX_TILING` | `57` |

| org.proteinshader.graphics.shader.ShaderProgramFactory | | |
| --- | --- | --- |
| `public static final String` | `SHADER_DIRECTORY` | `"./../shaders/"` |

| org.proteinshader.graphics.textures.TextureFactory | | |
| --- | --- | --- |
| `public static final String` | `BEND_CONFIG` | `"bend_textures.conf"` |
| `public static final String` | `BEND_DIRECTORY` | `"./../textures/bend/"` |
| `public static final String` | `HALFTONING_CONFIG` | `"halftoning_textures.conf"` |
| `public static final String` | `HALFTONING_DIRECTORY` | `"./../textures/halftoning/"` |
| `public static final String` | `PATTERNS_CONFIG` | `"patterns_textures.conf"` |
| `public static final String` | `PATTERNS_DIRECTORY` | `"./../textures/patterns/"` |

| org.proteinshader.graphics.typography.GLF2GlyphSetFactory | | |
| --- | --- | --- |
| `public static final int` | `BYTES_PER_PIXEL` | `2` |
| `public static final int` | `DATA_TYPE` | `5121` |
| `public static final int` | `FORMAT` | `6410` |

| org.proteinshader.graphics.typography.GLF2Header | | |
| --- | --- | --- |
| `public static final int` | `BYTES_PER_PIXEL` | `2` |
| `public static final int` | `CHAR_INFO_BYTES` | `24` |
| `public static final int` | `END_INDEX` | `16` |
| `public static final int` | `HEADER_BYTES` | `24` |
| `public static final int` | `HEIGHT_INDEX` | `8` |
| `public static final int` | `START_INDEX` | `12` |
| `public static final int` | `WIDTH_INDEX` | `4` |
| `public static final int` | `X1_INDEX` | `8` |
| `public static final int` | `X2_INDEX` | `16` |
| `public static final int` | `Y1_INDEX` | `12` |
| `public static final int` | `Y2_INDEX` | `20` |

| org.proteinshader.graphics.typography.GlyphSetFactoryFactory | | |
| --- | --- | --- |
| `public static final String` | `FONT_DIRECTORY` | `"./../fonts/"` |

| org.proteinshader.graphics.typography.TextLabel | | |
| --- | --- | --- |
| `public static final int` | `HEIGHT` | `32` |
| `public static final int` | `WIDTH` | `128` |

| org.proteinshader.graphics.typography.TextLabelManager | | |
| --- | --- | --- |
| `public static final int` | `BYTES_PER_PIXEL` | `2` |
| `public static final String` | `MAP` | `"textLabelMap"` |
| `public static final int` | `TABLA_RASA_HEIGHT` | `128` |
| `public static final int` | `TABLA_RASA_WIDTH` | `64` |
| `public static final int` | `X_OFFSET` | `15` |
| `public static final int` | `Y_OFFSET` | `0` |

| org.proteinshader.gui.ProteinShaderGUI | | |
| --- | --- | --- |
| `public static final int` | `BOTTOM_MARGIN` | `60` |
| `public static final int` | `TOP_MARGIN` | `30` |

| org.proteinshader.gui.ProteinShaderSplasher | | |
| --- | --- | --- |
| `public static final String` | `IMAGE_FILE` | `"ProteinShaderLogo.png"` |
| `public static final long` | `MIN_SPLASH_TIME` | `5000L` |

| org.proteinshader.gui.components.SpringLoadedJFrame | | |
| --- | --- | --- |
| `public static final double` | `DEFAULT_LONG_FACTOR` | `0.9` |
| `public static final double` | `DEFAULT_SHORT_FACTOR` | `0.5` |
| `public static final int` | `DELAY` | `10` |
| `public static final double` | `INCREMENT` | `0.05` |
| `public static final double` | `MAX_HEIGHT_FACTOR` | `0.9` |
| `public static final double` | `MAX_WIDTH_FACTOR` | `0.9` |
| `public static final double` | `MIN_HEIGHT_FACTOR` | `0.1` |
| `public static final double` | `MIN_WIDTH_FACTOR` | `0.1` |

| org.proteinshader.gui.components.controlpanel.AntialiasingPanel | | |
| --- | --- | --- |
| `public static final String` | `MENU_NAME` | `"Antialiasing"` |

| org.proteinshader.gui.components.controlpanel.AtomColorPanel | | |
| --- | --- | --- |
| `public static final String` | `MENU_NAME` | `"Atom Color"` |

| org.proteinshader.gui.components.controlpanel.AtomScalePanel | | |
| --- | --- | --- |
| `public static final String` | `MENU_NAME` | `"Atom Scale"` |
| `public static final double` | `SCALE_INCREMENT` | `0.1` |
| `public static final double` | `SCALE_INIT` | `1.0` |
| `public static final double` | `SCALE_MAX` | `10.0` |
| `public static final double` | `SCALE_MIN` | `0.1` |

| org.proteinshader.gui.components.controlpanel.AtomVisibilityPanel | | |
| --- | --- | --- |
| `public static final String` | `MENU_NAME` | `"Atom Visibility"` |

| org.proteinshader.gui.components.controlpanel.CartoonColorPanel | | |
| --- | --- | --- |
| `public static final String` | `MENU_NAME` | `"Cartoon Color"` |

| org.proteinshader.gui.components.controlpanel.CartoonSideChainsPanel | | |
| --- | --- | --- |
| `public static final String` | `MENU_NAME` | `"Cartoon Side Chains"` |

| org.proteinshader.gui.components.controlpanel.CartoonVisibilityPanel | | |
| --- | --- | --- |
| `public static final String` | `MENU_NAME` | `"Cartoon Visibility"` |

| org.proteinshader.gui.components.controlpanel.DecorationsPanel | | |
| --- | --- | --- |
| `public static final String` | `MENU_NAME` | `"Decorations"` |

| org.proteinshader.gui.components.controlpanel.MotionPanel | | |
| --- | --- | --- |
| `public static final int` | `MAX_SPEED` | `90` |
| `public static final String` | `MENU_NAME` | `"Motion"` |
| `public static final int` | `MIN_SPEED` | `-90` |
| `public static final int` | `X_SPEED_INIT` | `0` |
| `public static final int` | `Y_SPEED_INIT` | `5` |

| org.proteinshader.gui.components.controlpanel.OptimizePanel | | |
| --- | --- | --- |
| `public static final String` | `MENU_NAME` | `"Tiling"` |
| `public static final int` | `SPINNER_MAX` | `100` |
| `public static final int` | `SPINNER_MIN` | `3` |

| org.proteinshader.gui.components.controlpanel.SelectorPanel | | |
| --- | --- | --- |
| `public static final int` | `DIALOG_HEIGHT` | `300` |
| `public static final int` | `DIALOG_WIDTH` | `150` |

| org.proteinshader.gui.components.controlpanel.VisibilityPanel | | |
| --- | --- | --- |
| `public static final int` | `SLIDER_INIT` | `75` |

| org.proteinshader.gui.components.menubar.AboutBox | | |
| --- | --- | --- |
| `public static final int` | `EXTRA_HEIGHT` | `90` |
| `public static final int` | `EXTRA_WIDTH` | `40` |

| org.proteinshader.gui.components.menubar.FileMenu | | |
| --- | --- | --- |
| `public static final String` | `DATA_DIRECTORY` | `"./../data"` |
| `public static final String` | `IMAGES_DIRECTORY` | `"./../images"` |

| org.proteinshader.gui.components.menubar.HelpMenu | | |
| --- | --- | --- |
| `public static final String` | `HELP_FILE` | `"../help/index.html"` |
| `public static final String` | `IMAGE_FILE` | `"ProteinShaderLogo.png"` |
| `public static final String` | `SOURCEFORGE_WEB_SITE` | `"http://proteinshader.sourceforge.net"` |

| org.proteinshader.gui.listeners.CanvasMouseListener | | |
| --- | --- | --- |
| `public static final int` | `B1_MASK` | `16` |
| `public static final int` | `B2_MASK` | `8` |
| `public static final int` | `B3_MASK` | `4` |
| `public static final int` | `CAMERA_XY_SCALE` | `2` |
| `public static final int` | `CAMERA_ZOOM_SCALE` | `4` |
| `public static final int` | `WHEEL_SCALE_FACTOR` | `2` |

| org.proteinshader.gui.utils.FileExtensionFilter | | |
| --- | --- | --- |
| `public static final String` | `DEFAULT_EXTENSION` | `".txt"` |

| org.proteinshader.gui.utils.FpsLabel | | |
| --- | --- | --- |
| `public static final String` | `FONT_DIRECTORY` | `"./../fonts/"` |
| `public static final String` | `FONT_FILE` | `"ArialBold18.glf"` |

| org.proteinshader.gui.utils.ImageFileChooser | | |
| --- | --- | --- |
| `public static final String` | `DEFAULT_FILENAME` | `"canvas"` |

| org.proteinshader.gui.viewing.Camera | | |
| --- | --- | --- |
| `public static final double` | `FOVY` | `60.0` |
| `public static final double` | `HALF_FOVY` | `30.0` |
| `public static final double` | `HALF_FOVY_RADIANS` | `0.5235987755982988` |
| `public static final int` | `MIN_INITIAL_CAMERA_DISTANCE` | `20` |

| org.proteinshader.gui.viewing.Rotation | | |
| --- | --- | --- |
| `public static final double` | `HALF_PI` | `1.57079632679489` |
| `public static final double` | `PI` | `3.14159265358979` |
| `public static final double` | `RADIANS_PER_DEGREE` | `0.017453292519943278` |
| `public static final double` | `TWO_PI` | `6.28318530717958` |

| org.proteinshader.math.HermiteDemo | | |
| --- | --- | --- |
| `public static final int` | `SEGMENTS` | `10` |

| org.proteinshader.math.LocalFrameDemo | | |
| --- | --- | --- |
| `public static final int` | `BANNER_WIDTH` | `70` |

| org.proteinshader.math.Point3d | | |
| --- | --- | --- |
| `public static final String` | `DECIMAL_FORMAT` | `"0.000"` |

| org.proteinshader.math.Quaternion | | |
| --- | --- | --- |
| `public static final String` | `DECIMAL_FORMAT` | `"0.000"` |
| `public static final double` | `MIN_THETA` | `0.01` |
| `public static final double` | `ONE_DEGREE` | `0.017453292519943295` |

| org.proteinshader.math.QuaternionDemo | | |
| --- | --- | --- |
| `public static final int` | `BANNER_WIDTH` | `70` |

| org.proteinshader.math.SlerpDemo | | |
| --- | --- | --- |
| `public static final int` | `BANNER_WIDTH` | `72` |

| org.proteinshader.math.Vec3d | | |
| --- | --- | --- |
| `public static final String` | `DECIMAL_FORMAT` | `"0.000"` |

| org.proteinshader.structure.Drawable | | |
| --- | --- | --- |
| `public static final float` | `DEFAULT_ALPHA` | `1.0f` |
| `public static final float` | `DEFAULT_BLUE` | `0.0f` |
| `public static final float` | `DEFAULT_GREEN` | `0.0f` |
| `public static final double` | `DEFAULT_RADIUS` | `1.0` |
| `public static final float` | `DEFAULT_RED` | `0.0f` |
| `public static final float` | `DEFAULT_SPECULAR_EXP` | `20.0f` |

| org.proteinshader.structure.Heterogen | | |
| --- | --- | --- |
| `public static final String` | `HETEROGEN_PREFIX` | `"HET_"` |

| org.proteinshader.structure.Segment | | |
| --- | --- | --- |
| `public static final double` | `MAX_BEND` | `0.59` |

| org.proteinshader.structure.Structure | | |
| --- | --- | --- |
| `public static final int` | `SERIAL_NO_CACHE_CAPACITY` | `2000` |

| org.proteinshader.structure.enums.AtomEnum | | |
| --- | --- | --- |
| `public static final double` | `BALL_SCALE` | `0.2` |

| org.proteinshader.structure.factory.BetaStrandSegmentFactory | | |
| --- | --- | --- |
| `public static final double` | `HALF_PI` | `1.5707963267948966` |

| org.proteinshader.structure.factory.SegmentFactory | | |
| --- | --- | --- |
| `public static final double` | `MAX_CA_DISTANCE` | `4.5` |
| `public static final double` | `SEGMENT_LENGTH` | `2.6` |

| org.proteinshader.structure.io.PDBLineParser | | |
| --- | --- | --- |
| `public static final String` | `DEFAULT_CHAIN_ID` | `"A"` |
| `public static final String` | `DEFAULT_MODEL_ID` | `"1"` |
| `public static final int` | `MIN_CHAR_PER_LINE` | `80` |

| org.proteinshader.structure.io.PDBStructureReader | | |
| --- | --- | --- |
| `public static final int` | `MIN_CHAR_IN_HEADER` | `80` |

| org.proteinshader.structure.io.TubeVertices | | |
| --- | --- | --- |
| `public static final double` | `ALPHA_RADIUS` | `1.5` |
| `public static final int` | `ALPHA_SLICES` | `20` |
| `public static final int` | `ALPHA_STACKS` | `10` |
| `public static final double` | `BETA_RADIUS` | `1.5` |
| `public static final int` | `BETA_SLICES` | `20` |
| `public static final int` | `BETA_STACKS` | `10` |
| `public static final double` | `HALF_PI` | `1.57079632679489` |
| `public static final double` | `LOOP_RADIUS` | `0.75` |
| `public static final int` | `LOOP_SLICES` | `10` |
| `public static final int` | `LOOP_STACKS` | `10` |
| `public static final double` | `PI` | `3.14159265358979` |
| `public static final double` | `TWO_PI` | `6.28318530717958` |

| org.proteinshader.structure.io.demos.PDBStructureReaderDemo | | |
| --- | --- | --- |
| `public static final String` | `PATH` | `"edu/harvard/fas/jrweber/molecular/structure/io/demos/"` |
| `public static final String` | `usageMessage` | `"USAGE: java -cp . edu/harvard/fas/jrweber/molecular/structure/io/demos/PDBStructureReaderDemo inputFilename outputFilename"` |

| org.proteinshader.structure.io.demos.SegmentDemo | | |
| --- | --- | --- |
| `public static final String` | `PATH` | `"edu/harvard/fas/jrweber/molecular/structure/io/demos/"` |
| `public static final String` | `usageMessage` | `"USAGE: java -cp . edu/harvard/fas/jrweber/molecular/structure/io/demos/SegmentDemo inputFilename outputFilename"` |

| org.proteinshader.structure.visitor.BondPredictor | | |
| --- | --- | --- |
| `public static final double` | `DEFAULT_MIN_BOND_LENGTH` | `0.4` |
| `public static final double` | `DEFAULT_TOLERANCE` | `0.56` |

| org.proteinshader.structure.visitor.FrenetFrameGeneratorVisitor | | |
| --- | --- | --- |
| `public static final double` | `MAX_CA_DISTANCE` | `4.5` |

| org.proteinshader.structure.visitor.SFWriterVisitor | | |
| --- | --- | --- |
| `public static final int` | `CHAR_PER_LINE` | `80` |
| `public static final int` | `INDENTATION_FACTOR` | `2` |

| org.proteinshader.structure.visitor.demos.ModifierVisitorDemo | | |
| --- | --- | --- |
| `public static final String` | `PATH` | `"edu/harvard/fas/jrweber/molecular/structure/visitor/demos/"` |
| `public static final String` | `usageMessage` | `"USAGE: java -cp . edu/harvard/fas/jrweber/molecular/structure/visitor/demos/ModifierVisitorDemo inputFilename outputFilename"` |

| org.proteinshader.structure.visitor.demos.VisitorDemo | | |
| --- | --- | --- |
| `public static final String` | `PATH` | `"edu/harvard/fas/jrweber/molecular/structure/visitor/demos/"` |
| `public static final String` | `usageMessage` | `"USAGE: java -cp . edu/harvard/fas/jrweber/molecular/structure/visitor/demos/VisitorDemo filename"` |

---


|  |  |  |
| --- | --- | --- |
| **Overview** \| Package \| Class \| Use \| **Tree** \| **Deprecated** \| **Index** \| **Help** | |  |
| PREV   NEXT | **FRAMES**    **NO FRAMES**     **All Classes** |  |


---

# *Copyright © 2007-2008*
